# Different Effects of Androgen on the Expression of *Fut1*, *Fut2*, *Fut4* and *Fut9* in Male Mouse Reproductive Tract

**DOI:** 10.3390/ijms141123188

**Published:** 2013-11-21

**Authors:** Chun-Mei Wang, Shuang-Gang Hu, Yan-Fei Ru, Guang-Xin Yao, Wu-Bin Ma, Yi-Hua Gu, Chen Chu, Shou-Lin Wang, Zuo-Min Zhou, Qiang Liu, Yu-Chuan Zhou, Yong-Lian Zhang

**Affiliations:** 1 Shanghai Key Laboratory of Molecular Andrology, State Key Laboratory of Molecular Biology, Institute of Biochemistry and Cell Biology, Shanghai Institute for Biological Sciences, Chinese Academy of Sciences, Shanghai 200031, China; E-Mails: cmwang@sibcb.ac.cn (C.-M.W.); sghu@sibs.ac.cn (S.-G.H.); ruyanfei@sibcb.ac.cn (Y.-F.R.); ygx850226@163.com (G.-X.Y.); mawubin@sibcb.ac.cn (W.-B.M.); yhgu@sibs.ac.cn (Y.-H.G.); chuchen@sibcb.ac.cn (C.C.); qliu@sibs.ac.cn (Q.L.); 2 State Key Laboratory of Reproductive Medicine, Institute of Toxicology, School of Public Health, Nanjing Medical University, Nanjing 211166, China; E-Mail: wangshl@njmu.edu.cn; 3 State Key Laboratory of Reproductive Medicine, Department of Histology and Embryology, Nanjing Medical University, Nanjing 210029, China; E-Mail: zhouzm@njmu.edu.cn; 4 Shanghai Institute of Planned Parenthood Research, Shanghai 200032, China

**Keywords:** fucosyltransferase, androgen, male reproductive tract

## Abstract

The α-(1,2) fucosyltransferases (*Fut1* and *Fut2*) and α-(1,3) fucosyltransferases (*Fut4*, *Fut9*) are responsible for the synthesis of Lewis X (LeX) and Lewis Y (LeY) conjugated to glycoproteins. We recently reported that these fucosyltransferases were differentially expressed in the reproductive tract of male mouse. Here, we studied the effect of androgen on fucosyltransferase expression through the use of mouse castration models. We found that *Fut1* mRNA and *Fut4* mRNA were upregulated, while *Fut2* mRNA and *Fut9* mRNA were downregulated by androgen in the caput epididymis. However, in the vas deferens and prostate, only *Fut4* mRNA and *Fut2* mRNA were respectively upregulated following exposure to androgen. In the seminal vesicle, all fucosyltransferases, with the exception of *Fut9*, were upregulated. We identified the androgen receptor binding sites (ARBSs) of *Fut2*, *Fut4* and *Fut9* in the caput epididymis. Luciferase assay for these ARBSs is able to provide an indication as to why *Fut4* and *Fut9* are differently expressed and regulated by androgen, although they catalyze the same α-(1,3) fucose linkage. Our study showed that androgen could differentially regulate the expression of these fucosyltransferases and provided an insight into the characteristic distribution of each fucosyltransferase responsible for LeX/LeY biosynthesis in the male reproductive tract.

## 1. Introduction

Fucosylation is a common type of glycosylation of proteins. In mammals, fucose-containing glycans, such as Lewis antigens, have important roles in blood transfusion reactions, selectin mediated leukocyte-endothelial adhesion, host-microbe interactions, fertility and numerous ontogenic events [1]. Fucosyltransferases are the enzymes responsible for the catalysis of fucose transfer from donor guanosine-diphosphate (GDP) fucose to various acceptor molecules, including oligosaccharides, glycoproteins and glycolipids [2]. In the mouse, the functional α-(1,2) fucosyltransferases for LeY synthesis include *Fut1* and *Fut2* [3]. The functional α-(1,3) fucosyltransferases for LeX synthesis include *Fut4* [4] and *Fut9* [5]. *Fut7* can only synthesize sialylated LeX [6], while the catalytic activity of *Fut11*, a novel α-(1,3) fucosyltransferase, is only verified in human, so far [7].

The changing microenvironment of the epididymis, including glycoproteins secreted from the epithelium, is important for the maturation and fertilizing capacity of mammalian spermatozoa. Our previous study showed that α-(1,3) fucosyltransferases (*Fut4*, *Fut9* and *Fut11*) and α-(1,2) fucosyltransferases (*Fut1* and *Fut2*) were differently expressed and controlled the biosynthesis of LeX and LeY glycoproteins in the male mouse reproductive tract. Only *Fut1* and *Fut4* were highly expressed in zone 2 of caput epididymis and were found to be mainly responsible for the control of the LeY and/or LeX synthesis [8].

The gene expression and function in epididymis is greatly influenced by the luminal microenvironment [9]. Many genes in the efferent duct, initial segment and caput epididymis are regulated by testicular factors [10] and androgen [11]. However, it is not clear whether these fucosyltransferases were androgen-dependent. Previously, we have globally mapped the androgen receptor binding sites (ARBSs) by chromatin immunoprecipitation sequencing (ChIP-seq) in the mouse caput epididymis [12] in which the AR is highly expressed [13]. Through the ARBS library, we predicted the ARBSs that were located near the *Fut2*, *Fut4* and *Fut9* genes in the genome.

In the present investigation, we aimed to determine whether androgen regulates the expression and specific distribution of each fucosyltransferase in the mouse male reproductive tract and how these fucosyltransferases are regulated by androgen. The results of this study will help to understand the influence of androgen on the protein fucosylation and related pathology process in the male reproductive tract.

## 2. Results and Discussion

### 2.1. *Fut1* and *Fut4* mRNAs Are Upregulated, while *Fut2* and *Fut9* mRNAs Are Downregulated by Androgen in the Mouse Caput Epididymis

**Figure 1:**
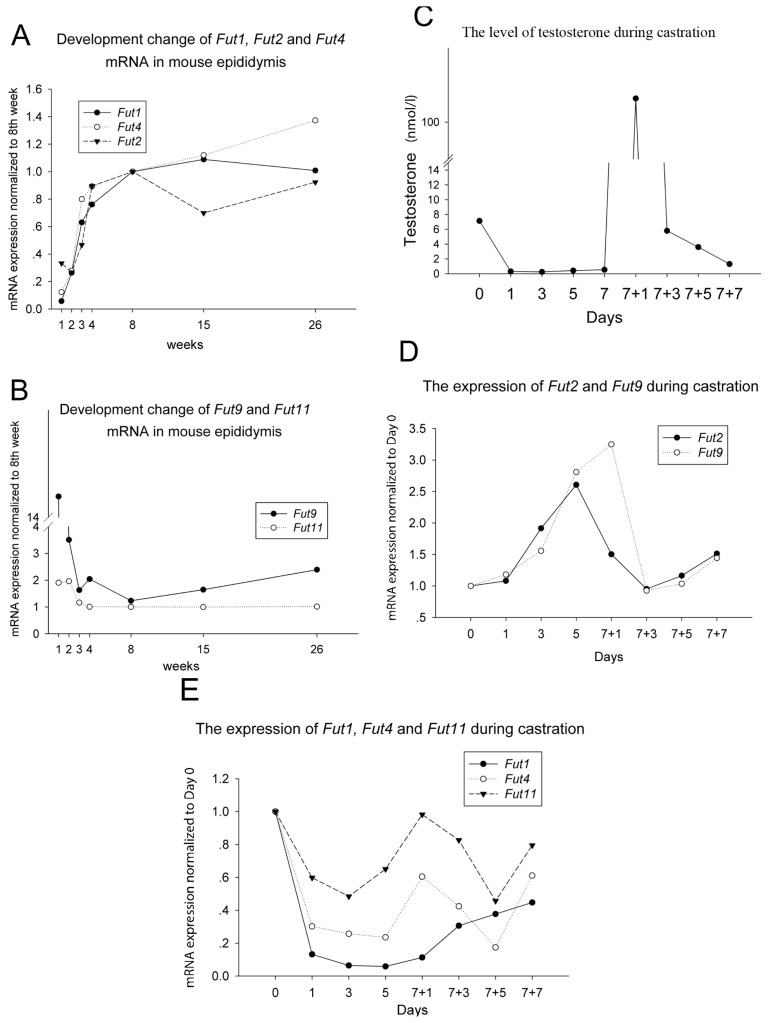
The mRNA expression of (**A**) *Fut1*, *Fut2* and *Fut4* and (**B**) *Fut9* and *Fut11* during mouse development. *X*-axis: age, weeks after birth. *Y*-axis: mRNA expression quantified by real time PCR, normalized to the eight weeks; (**C**) The change of serum testosterone concentrations following castration and androgen replacement. The expression of (**D**) *Fut2* and *Fut9* mRNA and (**E**) *Fut1*, *Fut4* and *Fut11* mRNA, following castration and androgen replacement. Three to five mice were used for each time point, and 3–5 pairs of epididymis were pooled together for RNA extract and RT-qPCR. qPCR for each sample were repeated at least three times. ‘//’ in the *Y* axis of (**B**) and (**C**) indicate a break to shrink the range of the column.

The epididymis is an androgen-responsive organ. Moreover, the greatest number of androgen-regulated genes were observed in the caput region when compared with regulation in either the corpus or cauda regions [14]. In addition, a gradual increase in serum androgen concentrations was observed from birth to sexual maturity in the mouse [15]. Here, the developmental changes on the expression of *Fut* mRNAs in the mouse caput epididymis throughout the life-span were surveyed by qPCR. The results indicated that the expression of *Fut1*, *Fut2* and *Fut4* rapidly increased after birth and continued until sexual maturation at eight weeks of age (Figure 1A). However, *Fut9* dramatically decreased after birth, and there was no significant change in *Fut11* expression during development (Figure 1B). The profile of serum androgen level during development [15] is the same as the change of *Fut1*, *Fut2* and *Fut4* and opposite to *Fut9*. Therefore, we hypothesized that these fucosyltransferases might be closely related to androgen regulation.

To investigate how these fucosyltransferases were regulated by androgen, we performed a time-curve castration model. In castrated animals, serum testosterone declined rapidly within one day and remained at low levels thereafter. On day 7 following castration, the administration of testosterone propionate resulted in a rapid increase in the serum testosterone concentration within 24 h. Testosterone concentration decreased to baseline levels on the third day after testosterone replacement and continued to decrease in the following days (Figure 1C). We found that *Fut2* and *Fut9* mRNA levels were increased after castration and were subsequently reduced following testosterone replacement, reaching the baseline level on the third day after injection. Then, a gradual increase in mRNA level was observed at the 7 + 5 days and 7 + 7 days following treatment (Figure 1D). This pattern was opposite to the observed change in serum testosterone concentration (Figure 1C). Conversely, *Fut1* and *Fut4* mRNA rapidly decreased after castration (Figure 1E), which mirrored the significant decrease of testosterone (Figure 1C). *Fut11* expression decreased rapidly, but rose without the androgen supplement on day 5, and went up and down again after androgen replacement. Its fluctuation was not in agreement with the change of testosterone level.

Taken together, in the mouse caput epididymis, androgen can positively regulate the expression of *Fut1* and *Fut4*, but negatively regulate the expression of *Fut2* and *Fut9*.

### 2.2. The Expression of *Fut1*, *Fut2*, *Fut4* and *Fut9* Are Regulated Differently by Androgen in Other Organs of the Male Mouse Reproductive Tract

How is the expression of *Fut1*, *Fut2*, *Fut4* and *Fut9* regulated by androgen in the tissues other than the epididymis? Here, we profiled the expression of these fucosyltransferases in these tissues in the “7 days castration + 2 days androgen replacement” castration model.

**Figure 2:**
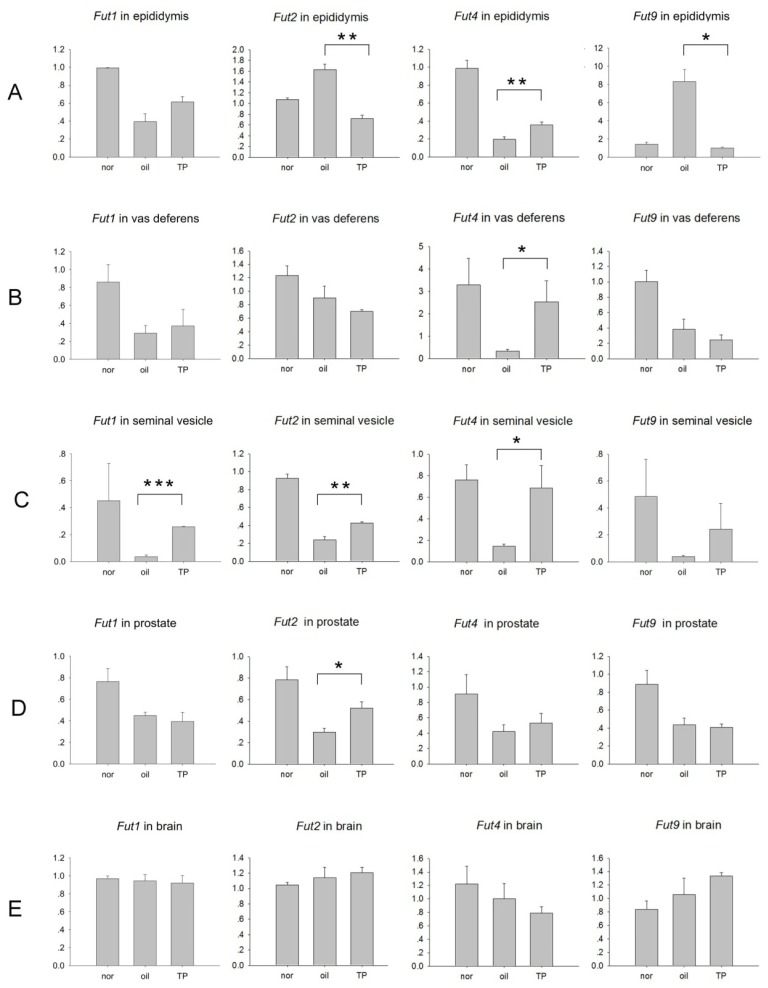
The expression of *Fut1*, *Fut2*, *Fut4* and *Fut9* in (**A**) epididymis; (**B**) vas deferens; (**C**) seminal vesicle; (**D**) prostate and (**E**) brain from animals in the “7 days castration + 2 days androgen replacement” castration model. Nor: normal non-surgery control; oil: oil vector control; TP: testosterone propionate replacement. *Y*-axis: mRNA expression of indicated genes using glyceraldehyde phosphate dehydrogenase (GAPDH) as the internal control. Data are expressed as the means ± SEM (*n* = 3). ^*^p < 0.05; ^**^p < 0.01; ^***^p < 0.001, as compared with the corresponding oil-treated castrated control.

In the epididymis, compared to the un-castrated normal mice group, *Fut1* mRNA and *Fut4* mRNA significantly decreased, while *Fut2* mRNA and *Fut9* mRNA significantly increased after castration in the oil control group. Compared to the oil control group, *Fut4* mRNA significantly increased, while *Fut2* mRNA and *Fut9* mRNA significantly decreased after testosterone propionate (TP) replacement, but the increase of *Fut1* mRNA after TP replacement was not significant (Figure 2A). This result is consistent to the time-curve model, which double confirmed both of the two models.

The expression of both *Fut4* in the vas deferens (Figure 2B) and *Fut2* in the prostate (Figure 2D) significantly decreased after castration in the oil control group and significantly increased after TP replacement, while the other three *Futs* were androgen-independent, respectively, in these two organs. In the seminal vesicle, except *Fut9*, all of the *Futs* showed a significant decrease after castration in the oil control group and a significant increase after TP replacement (Figure 2C); in brain, a non-typical androgen-dependent organ, there were no significant changes in *Fut* expression (Figure 2E).

Thus, *Fut1*, *Fut2*, *Fut4* and *Fut9* were differently expressed in the male reproductive tract, and they are also differentially regulated by androgen in each tissue of the male reproductive tract.

### 2.3. The AR-ARBS Binding Was Not the Only Reason for the Difference of Androgen Regulation between Epididymis and Prostate

**Figure 3:**
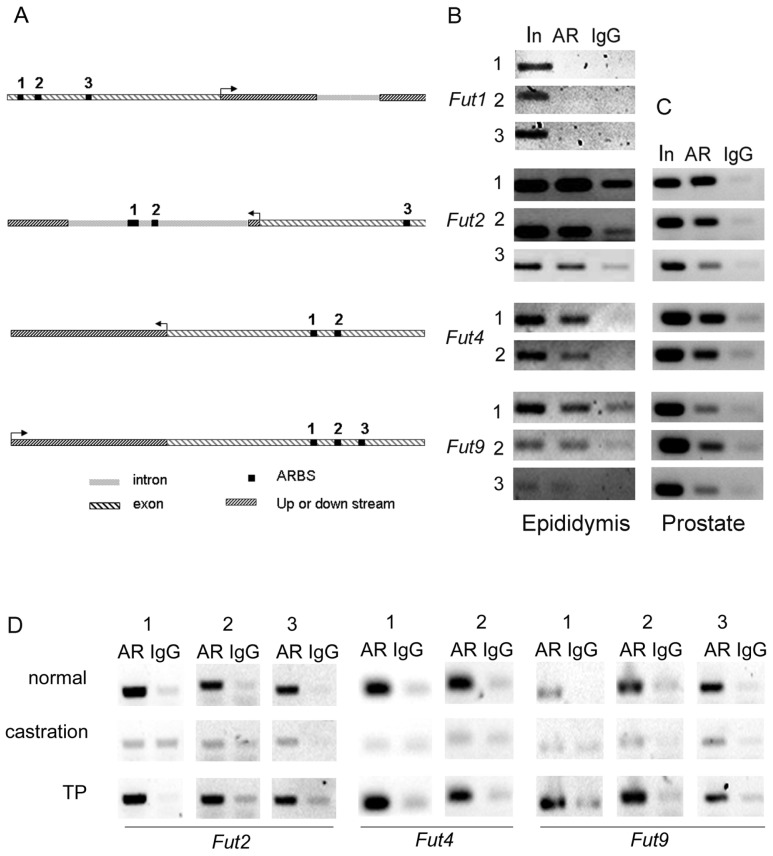
(**A**) The localization of the predicted androgen receptor binding sites (ARBSs) of *Fut1* and the ARBSs of *Fut2, Fut4* and *Fut9* retrieved from a previously developed chromatin immunoprecipitation sequencing (ChIP-seq) data library; The binding of AR to the ARBSs from the *Futs* were compared between (**B**) the caput epididymis and (**C**) the prostate by ChIP-PCR with AR antibody; (**D**) The binding of ARBSs to AR in control (normal), castrated and following testosterone propionate (TP) replacement treatment in the caput epididymis. 1, 2, 3 in (**B**), (**C**), (**D**) refer to the binding sites indicated in (**A**). AR: ChIP-PCR with AR antibody; IgG: ChIP-PCR with normal immunoglobulin G (IgG) as negative control.

Following androgen administration, the expression of *Fut2* was downregulated in the epididymis and upregulated in the prostate when compared to the castrated oil-treated group. In addition, upregulation of *Fut4* expression and downregulation of *Fut9* expression in epididymis was observed in the TP group, while there was no impact in the prostate (Figure 2). To determine if fucosyltransferases were directly regulated by androgen and if fucosyltransferases differentially alter AR regulation in different reproductive tissues, the ARBSs of fucosyltransferase genes were examined. In a previous investigation, we mapped the ARBSs in mouse epididymis *in vivo* through the use of chromatin immunoprecipitation sequencing (ChIP-seq) [12]. Using this library, we were able to locate potential ARBSs of *Fut2*, *Fut4* and *Fut9* (Figure 3A). Although results from developmental and castration experiments suggest that *Fut1* is upregulated by androgen, no ARBSs were found to be located around the *Fut1* gene in the genome. In order to rule out a possible false-negative finding, we investigated the region around the *Fut1* gene, from 5 kb upstream to 5 kb downstream using Genomatix Region Miner release 3.2 [16]. The results from this search predicted three potential ARBSs (Figure 3A), which are conserved in humans, rats and mice. We performed a ChIP-PCR with primers for about 150 bp around the ARBS to confirm the AR-binding characteristics of these ARBSs in the epididymis. The three predicted ARBSs in *Fut1* were shown to be negative, while the other ARBSs in *Fut2*, *Fut4* and *Fut9* from the library were immunoprecipitated by the AR, but not the immunoglobulin G (IgG) control (Figure 3B).

To examine if the binding of AR to the ARBS DNA sequences was triggered by androgen, we further performed ChIP-PCRs with or without testosterone stimulation. As expected, all the AR-ARBS binding in *Fut2*, *Fut4* and *Fut9* disappeared after castration and appeared again after the replacement of testosterone propionate (Figure 3D). Therefore the AR-ARBS binding in the epididymis was dependent on the stimulation of testosterone. Therefore, androgen can directly regulate *Fut2*, *Fut4* and *Fut9*.

However, the ChIP-PCR assay in prostate showed that the ARBSs identified in the caput epididymis could also bind to ARs in prostate (Figure 3C). Therefore, the AR-ARBS bindings were not the only reason responsible for the difference of AR regulation of *Fut2*, *Fut4* and *Fut9* between prostate and the caput epididymis.

### 2.4. Luciferase Assay Showed that Fut4 Was Upregulated by Androgen

**Figure 4:**
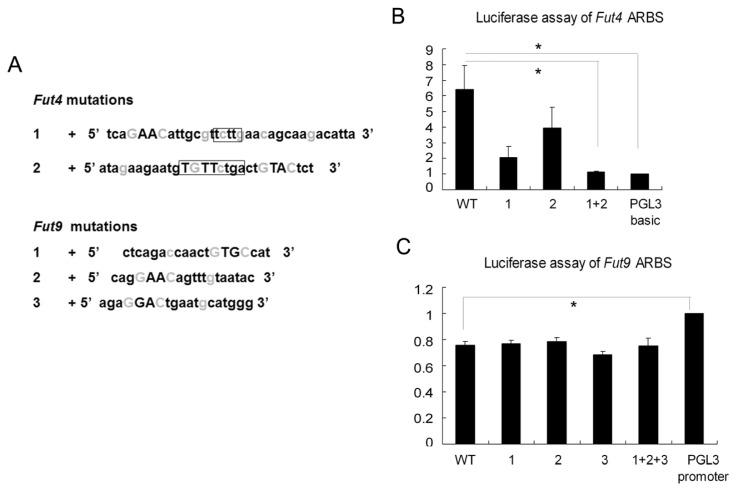
(**A**) The sequences and mutations of ARBSs of *Fut4* and *Fut9.* The key G/Cs in light gray were mutated to A/Ts. Capitalized letters indicate conserved core binding elements. In *Fut4*, the repeat sequences shared by the tandem ARBSs were labeled with boxes. Luciferase assay of (**B**) *Fut4* ARBSs and (**C**) *Fut9* ARBSs in response to the androgen stimulation. Data are expressed as the mean ± SEM (*n* = 3), ^*^p < 0.05, as compared with the corresponding control.

In addition to binding to ARBS, AR interacts with other cofactors to regulate the target genes [17]. To explore how *Fut4* and *Fut9* are differentially regulated by AR and cofactors in the epididymis and how they become androgen-independent in prostate, we would ideally compare the androgen-dependent characteristic of the ARBSs, especially the cofactors of the androgen receptor between *Fut4* and *Fut9*, and between the epididymis and prostate. Due to a lack of cell lines originating from epididymis and prostate, we used a Chinese hamster ovary (CHO) cell line to perform the luciferase assays. Two ARBSs in the *Fut4* AR-binding region and three ARBSs in the *Fut9* AR-binding region were retrieved from the library. We mutated the key G/Cs in the ARBS sequences into A/Ts to block the binding of the ARBS to AR (Figure 4A).

In *Fut4*, the response to androgen stimulation of the wild-type ARBSs was six times higher than that of the negative control vector. This promoter activity was not significantly blocked by the point mutation of either NO1 ARBS or NO2 ARBS individually. However, when both ARBS were mutated, the reaction was almost as weak as the negative control vector (Figure 4B). Therefore, the two ARBSs may work in reciprocal synergism. In *Fut9*, the response to androgen can be inhibited by the peak sequence containing the three ARBSs. However, none of the point mutations of the three ARBSs rescued this silencing activity (Figure 4C). This to some extent explained why in the male reproductive tract *Fut4* was upregulated in most of the organs, but *Fut9* was androgen-independent, except in the epididymis, although they catalyze the same α-(1,3) fucose linkage. The luciferase assays supported our opinion that testosterone was one important factor responsible for the dramatic difference of *Fut4* and *Fut9* expression in caput epididymis.

### 2.5. Discussion

The present study demonstrates for the first time that fucosyltransferases in the reproductive system are androgen-dependent and differentially regulated by androgen in the caput epididymis, vas deferens, seminal vesicle and prostate. However, androgen regulation cannot answer all the questions about the specific distribution of these fucosyltransferases. There may be other factors accounting for the regulation of these fucosyltransferases in the male reproductive tract.

The castration model showed that *Fut2* was downregulated by androgen in epididymis (Figure 2A), but it was consistent to the level of testosterone during development before eight weeks of age (Figure 1A). Before sexual maturation, other overwhelming factors may be responsible for this pattern of *Fut2* expression. In addition, *Fut2* was upregulated by androgen administration in the seminal vesicle and the prostate, an opposite response to that of the epididymis. Therefore, the regulation of *Fut2* expression appears to be time- and tissue-dependent.

The exact ARBSs of *Fut1* responsive to the androgen stimulation were not found in the current investigation. The slow recovery rate of *Fut1* in epididymis (Figure 1D) resulted in the non-significant increasing trend of *Fut1* mRNA after TP replacement in the “7 days + 2 days” castration model (Figure 2A). However, the change of *Fut1* expression was consistent with the level of androgen in both the developmental model and time-curve castration model of the epididymis (Figure 1A,C). The “7 days + 2 days” castration model also showed that *Fut1* was upregulated in the seminal vesicle (Figure 2C). It is possible that the ARBSs responsible for *Fut1* are too far away to be detected or, alternatively, that *Fut1* expression may be indirectly regulated by androgen in the caput epididymis.

Two castration models were used in the current study: a time-curve model and the “7 days + 2 days” castration model. Results from the time-curve model showed a change in gene expression levels throughout; however, a major drawback of this model is the lack of a true negative control, which is found in the “7 days + 2 days” castration model. Other factors that may have affected the results of these models must also be considered. *Fut4* mRNA was upregulated in castrated males following androgen administration in the epididymis (Figure 2A), vas deferens (Figure 2B) and seminal vesicle (Figure 2C). However, there was an unexpected increase in gene expression from day 5 to day 7 of androgen replacement after castration (Figure 1E). It has been documented that the epithelial cell of caput epididymis was significantly influenced and was going through inflammation and apoptosis at 12 days after removing the testis [18]. *Fut4* mRNA was reported to be increased in inflammation [19] and cell death [20,21]. Therefore it is possible that from the fifth day of androgen replacement, which is the 12th day after castration, the upregulation of *Fut4* mRNA by inflammation and apoptosis overwhelmed the downregulation of *Fut4* mRNA in response to the drop of androgen.

The diversity of androgen-dependent regulation of these fucosyltransferases may partially explain the characteristic distribution of each fucosyltransferase response for LeX/LeY biosynthesis in the male reproductive tract. In mouse caput epididymis, *Fut1* mRNA and *Fut4* mRNA are upregulated (Figure 1E), while *Fut2* mRNA and *Fut9* mRNA are downregulated (Figure 1D) by androgen. This different response to androgen may partially explain why *Fut1* and *Fut4* are highly expressed and control the expression of LeY and LeX in the caput epididymis, while *Fut2* and *Fut9* are not [8], although *Fut1* and *Fut2* play the same role in α(1,2) fucosylation, as well as *Fut4* and *Fut9* playing the same role in α(1,3) fucosylation. *Fut4* mRNA was upregulated by androgen in vas deferens and seminal vesicle (Figure 2B,C), which corresponds to the dominant expression of *Fut4* and LeX/LeY biosynthesis controlled by *Fut4* in these two organs [8]. *Fut2* mRNA was upregulated by androgen in seminal vesicle and prostate (Figure 2C,D); this is also in agreement with the dominant expression of *Fut2* and LeX/LeY biosynthesis controlled by *Fut2* in seminal vesicle and the coagulating gland (the anterior lobe of the prostate) [8].

In order to relate our findings to human investigations, it is necessary to consider that humans also express other fucosyltransferases (*Fut3*, *5* and *6*) compared to mice. These fucosyltransferases may also contribute to fucosylation in the male reproductive tract and may confound the contribution of either *Fut4* or *Fut9*. Further investigations are required to determine if *Fut3*, *Fut5* and *Fut6* expressions in the human reproductive tract are also androgen-dependent and differentially regulated.

## 3. Experimental Section

### 3.1. Animals

Male C57BL/6 mice were used in this study. The mice were housed in the Experimental Animal Center of the Chinese Academy of Science (Shanghai, China).

### 3.2. Mouse Development and Castration Model

In all animals, the epididymides were removed at 1, 2, 3, 4, 8, 15 and 26 weeks after birth.

The time-curve castration model for the epididymis and the “7 days + 2 days” castration models were conducted following a previously described protocol [14,22]. Briefly, 10-week-old adult male mice were castrated bilaterally under sodium pentobarbital anesthesia. For the time curve model of the epididymis, the mice were dividedly sacrificed either on days 0, 1, 3 and 5 after castration or 1, 3, 5 and 7 days after a single injection of testosterone propionate (5 μg/g body weight) applied to the 7th day castrated mice. For the “7 days + 2 days” castration model of the male reproductive tract, un-castrated mice were used as normal control, and bilaterally castrated mice were sacrificed on day 2 after a single injection of oil or testosterone propionate applied to the 7th day castrated mice, as the oil control group or TP treated group, respectively. Epididymis, vas deferens, seminal vesicle and all leaves of prostate (including the coagulating gland) were collected for RNA extraction.

For ChIP-PCR, castrated mice were killed at different times after castration (0 day and 3 days) or 2 days after two injections of testosterone propionate (2.5 mg/day) applied to the 3rd day castrated mice (3 days + 2 days), and caput epididymis of each group was pooled.

Testosterone content of pooled serum samples was measured by radioimmunoassay (RIA), and the epididymis samples for each group were pooled for RNA extraction.

### 3.3. RNA Preparation and Real-Time PCR

Total RNAs were extracted with Trizol (Invitrogen, Carlsbad, CA, USA), following the manufacturer’s recommendations. The reverse transcription reaction to synthesize cDNA was performed with a ReverTra Ace-α-TM kit (Toyobo Co., Osaka, Japan), according to the manufacturer’s instructions. The mRNA level of each *Fut* in different tissues was determined using real-time PCR with the Quantitect Synergy Brands (SYBR) Green PCR master mix (Qiagen, Valencia, CA, USA). The primers for α (1,2) *Fut* (*Fut1* and *Fut2*), α (1,3) *Fut* (*Fut4*, *Fut9* and *Fut11*) and GAPDH as an internal control are listed in Table 1.

### 3.4. AR-Binding Site Searching and ChIP-PCR

The AR-binding regions of fucosyltransferases were taken from the ChIP-seq data library [12]. Genomatix Region Miner release 3.2 [16] was used to map potential AR-binding sites (ARBSs) and, subsequently, the AR-binding regions around the fucosyltransferase.

ChIP assays were performed on the mouse caput epididymis and prostate as described [12]. Adult male mice were sacrificed, and the caput epididymis was excised and finely minced in phosphate buffer saline (PBS). After washing twice with PBS to remove epididymal lumen fluid and sperm, tissue was cross-linked in 1% formaldehyde at room temperature for 10 min. Tissue was pelleted, washed and re-suspended in PBS containing protease inhibitors and disaggregated on ice using 50 strokes of a glass homogenizer. Nuclei were collected and sonicated to yield 100–300 bp DNA fragments. Lysates were pre-cleared with Protein A beads (sc-2001, Santa Cruz Biotechnology, Santa Cruz, CA, USA) at 4 °C for 2 h before an overnight incubation with anti-AR (H-280, sc-13062, Santa Cruz Biotechnology, Santa Cruz, CA, USA) or normal rabbit IgG. After a short incubation with Protein A beads, chromatin-antibody-bead complexes were washed twice with low salt buffer containing 0.1% sodium dodecyl sulfate, 1% Triton X-100, 2 mM ethylene diamine tetraacetic acid (EDTA), 20 mM Tris-HCl (pH 8.1) and 0.15 M NaCl, twice with the same buffer, except containing 0.5 M NaCl, twice with lithium chloride buffer containing 0.25 M LiCl, 1% Nonidet P-40, 1% deoxycholate, 1mM EDTA and 10 mM Tris-HCl (pH 8.1) and three times with 1 mM EDTA and 10 mM Tris-HCl (pH 8.1). Chromatin was eluted with elution buffer containing 1% sodium dodecyl sulfate and 0.1 M NaHCO_3_ before reversal of the cross-links with Proteinase K (Invitrogen, Carlsbad, CA, USA) at 65 °C for 4 h. DNA was purified using two rounds of phenol-chloroform extraction and ethanol precipitation and resolved in optimal volume of double-distilled H_2_O. Anti-AR (H-280, sc-13062, Santa Cruz Biotechnology, Santa Cruz, CA, USA) and normal rabbit IgG (sc-2027) (Santa Cruz Biotechnology, Santa Cruz, CA, USA) were used for ChIP. DNA samples from ChIP preparations were analyzed by PCR (ChIP-PCR). Primers listed in Table 2 were used to amplify a 90- to 150-bp region around the ARBS.

### 3.5. Luciferase Reporter Assay

*Fut4* and *Fut9* AR-binding regions were cloned into plasmid with firefly luciferase (PGL3) basic and PGL3-promoter luciferase reporter vectors, respectively. Then, the key G/Cs in ARBS sequences were mutated to A/Ts. The primers for cloning and mutation are listed in Tables 3 and 4. CHO cells were grown in 96-well plates and co-transfected with 150 ng reporter constructs, 15 ng plasmid with renilla luciferase and thymidine kinase promoter (pRL-TK) and 15 ng pcDNA3.1-AR expression vector coding for full-length human AR by using Lipofectamine 2000 (Invitrogen, Carlsbad, CA, USA). After overnight transfection, cells were treated with 100 nM dihydrotestosterone (DHT) or ethanol vehicle for 24 h. Luciferase activities were measured using the dual-luciferase reporter assay (Promega, Madison, WI, USA) and a Mithras LB940 multimode microplate reader (Berthold, Bad Wildbad, Germany).

## 4. Conclusions

Our study showed that androgen could differentially regulate the expression of fucosyltransferases in the caput epididymis, vas deferens, seminal vesicle and prostate. These data may partially explain the characteristic distribution of each fucosyltransferase responsible for LeX/LeY biosynthesis in the male reproductive tract.

## Figures and Tables

**Table 1:** Primers for real time PCR of fucosyltransferases.

*Fut1*	sense: 5′ GCATCCGCCCTCATACCT 3′
	anti-sense: 5′ GCCAGCGAAGACCACATCA 3′

*Fut2*	sense: 5′ CCCACTTCCTCATCTTTGTCTTT 3′
	anti-sense: 5′ TTTGAACCGCCTGTAATTCCTT 3′

*Fut4*	sense: 5′ CAGCCTGCGCTTCAACATC 3′
	anti-sense: 5′ CGCCTTATCCGTGCGTTCT 3′

*Fut9*	sense: 5′ ATCCAAGTGCCTTATGGCTTCT 3′
	anti-sense: 5′ TGCTCAGGGTTCCAGTTACTCA 3′

*Fut11*	sense: 5′ TAACTTGGAAGACTGCGTTACTG 3′
	antisense: 5′ GGCTGAGATACTAGCTCCATACC 3′

*Gapdh*	sense: 5′ GGTGAAGCAGGCATCTGAGGG 3′
	anti-sense: 5′ GGTGGGTGGTCCAGGGTTT 3′

**Table 2:** Primers for ChIP-PCR.

*Fut1*	1 sense: 5′ AAAGAAGAAGAAGAAGAAGAAGAAG 3′
	anti-sense: 5′ CATTTTGGGCTCTGATAAAGCA 3′

	2 sense: 5′ AGGATGTTGCATCCTGGTTTGG 3′
	anti-sense: 5′ CTCTGTCCCACAGCCTCACTTTGA 3′

	3 sense: 5′ ACTCTAGATCTCATCCATTCCATCA 3′
	anti-sense: 5′ ACAGCCATTCACTTTGCCTGAG 3′

*Fut2*	1 sense: 5′ CCCTCTACCAAAGGAGCATTC 3′
	anti-sense: 5′ AACACCAAGTGGAGACGTTCAG 3′

	2 sense: 5′ AAACCTGCAATTCCAGCCAC 3′
	anti-sense: 5′ CGTGAATCTTGTGTGATGACCG 3′

	3 sense: 5′ TGTTATCCGGCCCATTGTGT 3′
	anti-sense: 5′ GGGTTACTGGAGCATAGCGC 3′

*Fut4*	1 sense: 5′ CGTGTGCTGGGATTACAGATACA 3′
	anti-sense: 5′ CAAGGACTTAACAAGGCAGGGG 3′

	2 sense: 5′ CTGCTTTGTGCTTTCTCTTTTGCT 3′
	anti-sense: 5′ ATATTCATTTTCCTGAACACCCAC 3′

*Fut9*	1 sense: 5′ GACTGCATGGAGCTCTCTGGAAG 3′
	anti-sense: 5′ AAGATAGCCACATAACCAAACCCA 3′

	2 sense: 5′ ATGTTGGCTTTGGTTCATGTCT 3′
	anti-sense: 5′ GCATTCAGTCCTCTGCTATTCAA 3′

	3 sense: 5′ TGTCTCCCAGGAACAGTTTGTAATA 3′
	anti-sense: 5′ CTTGCCAGCAGTAGTTTCCTATCA 3′

**Table 3:** Primers for sub-clones of *Fut4* and *Fut9* AR-binding regions.

*Fut4*	sense: 5′ CCAGGTACCTCTATTCTCTATTGCTAC 3′
	antisense: 5′ CAAGAGCTCAATCTCAGATTCCACT 3′
*Fut9*	sense: 5′ AGGTACCTCTCTGGAAGAAACAAAGA 3′
	antisense: 5′ ACCGAGCTCACGCATTTATTTTTAG 3′

**Table 4:** Primers for *Fut4* and *Fut9* mutations.

*Fut4*	1 sense	5′TACCTAGGTCCTGTAAGTCAACATAGCAATCAAAATATTGCTTTATTAAATAGCAATACATTACCCATTGAGTCATCTGGCC-3′
	antisense	5′GGCCAGATGACTCAATGGGTAATGTATTGCTATTTAATAAAGCAATATTTTGATTGCTATGTTGACTTACAGGACCTAGGTA-3′

	2 sense	5′TTCACTGACCTTAAGTTGGTACAGTCAATATAAGAATGTATTATGACTATATTCTTCCTTGAGTGAAGAGGTAATG-3′
	antisense	5′CATTACCTCTTCACTCAAGGAAGAATATAGTCATAATACATTCTTATATTGACTGTACCAACTTAAGGTCAGTGAA-3′

*Fut9*	1 sense	5′-GAGCTGTGAAAGAAGCTCAGATCAACTATGACATCTGAAAAGCACAGTCTTT-3′
	antisense	5′-AAAGACTGTGCTTTTCAGATGTCATAGTTGATCTGAGCTTCTTTCACAGCTC-3′

	2 sense	5′-ATGTTGGCTTTGGTTCATGTCTCCCAGTAAAAGTTTATAATACAATATAATTGGCAATTGAAATG-3′
	antisense	5′-CATTTCAATTGCCAATTATATTGTATTATAAACTTTTACTGGGAGACATGAACCAAAGCCAACAT-3′

	3 sense	5′-AATAGAACTAAAGTTGAATAGCAGATGAATGAATACATGGGTCTTGAAGCTATGTGTCTC-3′
	antisense	5′-GAGACACATAGCTTCAAGACCCATGTATTCATTCATCTGCTATTCAACTTTAGTTCTATT-3′
